# Bidirectional Interaction Between Liposomal Amphotericin B Pharmacokinetics and Parasite Dynamics in Patients With Post‐Kala‐Azar Dermal Leishmaniasis: Potential Implications for Optimal Dosing

**DOI:** 10.1002/cpt.70124

**Published:** 2025-11-25

**Authors:** Wan‐Yu Chu, Om Prakash Singh, Shyam Sundar, Dinesh Mondal, Krishna Pandey, Pradeep Das, Ignace C. Roseboom, Sheeraz Raja, Ana Torres, Eugenia Carrillo, Alwin D.R. Huitema, Fabiana Alves, Thomas P.C. Dorlo

**Affiliations:** ^1^ Department of Pharmacy Uppsala University Uppsala Sweden; ^2^ Department of Pharmacy and Pharmacology Netherlands Cancer Institute Amsterdam The Netherlands; ^3^ Department of Biochemistry, Institute of Science Banaras Hindu University Varanasi India; ^4^ Department of Medicine, Institute of Medical Sciences Banaras Hindu University Varanasi India; ^5^ Kala‐azar Medical Research Center (KARMC) Muzaffarpur India; ^6^ International Centre for Diarrhoeal Disease Research, (icddr,b) Dhaka Bangladesh; ^7^ ICMR Rajendra Memorial Research Institute of Medical Sciences (RMRIMS) Patna India; ^8^ Department of Molecular Parasitology ICMR‐National Institute for Research in Bacterial Infections Kolkata India; ^9^ Drugs for Neglected Diseases initiative (DNDi) New Delhi India; ^10^ WHO Collaborating Centre for Leishmaniasis, National Centre for Microbiology Instituto de Salud Carlos III Madrid Spain; ^11^ CIBER de Enfermedades Infecciosas Instituto de Salud Carlos III Madrid Spain; ^12^ Department of Pharmacology Princess Máxima Center for Pediatric Oncology Utrecht The Netherlands; ^13^ Department of Clinical Pharmacy University Medical Centre Utrecht, Utrecht University Utrecht The Netherlands; ^14^ Drugs for Neglected Diseases initiative (DNDi) Geneva Switzerland

## Abstract

Post‐kala‐azar dermal leishmaniasis (PKDL) involves a high macrophage burden in which the *Leishmania* parasites reside. Liposomal amphotericin B (LAmB) plays a key role in the treatment of PKDL. The mononuclear phagocyte system (MPS) is crucial in the distribution of liposomal drugs as well as the leishmaniasis pathophysiology. This study focused on characterizing the interaction between LAmB pharmacokinetics, the MPS, and parasite dynamics for optimal dosing of LAmB in PKDL. Clinical trial data from the Indian subcontinent, involving short‐course LAmB administered alone or with miltefosine, were analyzed using nonlinear mixed‐effects modeling. The pharmacokinetics of LAmB were best described by a two‐compartment model with a saturable LAmB uptake by the MPS. The maximum MPS uptake capacity was modeled with a baseline component and an additional disease‐related component relative to the parasite burden. As treatment progressed, MPS capacity decreased with declining parasite load, resulting in a median 54% increase in the systemic LAmB exposure (AUC_0‐24h_) by the end of treatment. Simulations suggested that a similar parasite clearance could be achieved with a 50% lower total LAmB dose, supporting the potential efficacy of reduced dosing regimens. Combining LAmB and miltefosine further accelerated parasite clearance compared to LAmB alone. This study highlights the importance of understanding the bidirectional interactions between LAmB pharmacokinetics and parasite infection for interpreting systemic exposure and optimizing treatment approaches. If confirmed in clinical trials, reduced LAmB dosing strategies could enable more rational and cost‐effective management of PKDL and other dermal leishmaniases.

**Study Highlights**

**WHAT IS THE CURRENT KNOWLEDGE ON THE TOPIC?**

The pharmacokinetics of liposomal amphotericin B (LAmB) has not been characterized in any form of human leishmaniasis. In fungal conditions, a nonlinear dose–exposure relationship for LAmB has been suggested, likely due to the saturable uptake of liposomes by the mononuclear phagocyte system (MPS), which is highly activated in patients with leishmaniasis.

**WHAT QUESTION DID THE STUDY ADDRESS?**

How do the pharmacokinetics of LAmB relate to *Leishmania* infection, and how can treatment regimens containing LAmB be optimized for post‐kala‐azar dermal leishmaniasis (PKDL)?

**WHAT DOES THIS STUDY ADD TO OUR KNOWLEDGE?**

Drug treatment reduced the parasite burden, which in turn affected the host response involving the MPS. Therefore, the capacity of the MPS to uptake LAmB decreased with the reduced parasite burden, leading to increased systemic exposure to LAmB over time.

**HOW MIGHT THIS CHANGE CLINICAL PHARMACOLOGY OR TRANSLATIONAL SCIENCE?**

First, the dose–response relationship of LAmB and other liposomal drugs cannot be solely determined by systemic exposure, particularly in diseases with a high macrophage burden, such as leishmaniasis. Second, the total LAmB dose used in PKDL could potentially be reduced by administering lower daily doses while maintaining the five administrations divided over a 15‐day treatment period. Lastly, combining miltefosine with LAmB achieved a greater reduction in parasite levels than treatment with LAmB alone.

Leishmaniasis is a neglected parasitic tropical disease, characterized by a large recruitment of macrophages in which *Leishmania* parasites reside and replicate.^1^ Post‐kala‐azar dermal leishmaniasis (PKDL) is a dermal complication following visceral leishmaniasis (VL) caused by *Leishmania donovani*.^2^ On the Indian subcontinent (ISC), 5–10% of VL cases develop PKDL within 2–3 years, primarily as macular lesions. These lesions may serve as reservoirs for parasites, perpetuating disease transmission and posing a public health challenge to VL elimination efforts.^3^, ^4^

Managing PKDL is complicated by uncertainties regarding optimal treatment regimens and durations, as well as the slow process of lesion repigmentation and healing. On the ISC, oral miltefosine administered over 3 months is the standard treatment for PKDL. However, this regimen is associated with poor adherence, increased risks of gastrointestinal and ocular toxicities, and contraception is required for 8 months.^5^ Therefore, recent clinical research has focused on shortened treatment options, including liposomal amphotericin B (LAmB) as monotherapy for 15 days or in combination with oral miltefosine for 21 days.^6^, ^7^, ^8^

LAmB, a unilamellar liposomal formulation of amphotericin B, demonstrates excellent efficacy against leishmaniasis, likely due to its selective uptake by the mononuclear phagocyte system (MPS), particularly by macrophages, as part of innate immunity. Therefore, using liposomes as delivery vehicles for antileishmanial drugs is thought to facilitate targeted drug delivery.^9^, ^10^, ^11^ Despite its potential, the pharmacokinetic‐pharmacodynamic (PK‐PD) relationships of LAmB, alone or combined with miltefosine, remain unexplored in any form of human leishmaniasis.^12^ The PK of liposomal drugs is inherently complex as MPS‐mediated phagocytosis strongly influences their disposition.^9^, ^10^ Processes such as opsonization and receptor saturation may lead to nonlinear dose‐exposure relationships,^13^, ^14^, ^15^ as evidenced by previous studies in fungal infections where more than dose‐proportional increases in exposure and substantial accumulation after repeated dosing have been reported.^16^, ^17^

Given a highly activated MPS and a higher macrophage burden in patients with leishmaniasis compared with other infectious diseases,^18^ it was hypothesized that parasite burden directly influences MPS uptake of LAmB.^19^, ^20^, ^21^ The aims of this study were to characterize the bidirectional interplay between LAmB distribution and *Leishmania* parasite infection, to understand how disease pathophysiology affects drug distribution, and to optimize LAmB treatment regimens for PKDL. To enable this, PK‐PD models were developed to describe the systemic PK of LAmB and miltefosine and their PD effects on *Leishmania* parasite clearance in patients with PKDL.

## METHODS

### Study design and participants

A non‐comparative, open‐label, randomized phase II clinical trial was conducted in Bangladesh and India to assess the safety and efficacy of short‐course treatment modalities for patients with PKDL on the ISC.^8^ The two treatment modalities investigated were LAmB monotherapy and a combination of LAmB with oral miltefosine. A secondary objective of this trial was to characterize the PK‐PD of LAmB and miltefosine in this population. The trial was registered with the Clinical Trials Registry—India (CTRI) under the reference number CTRI/2017/04/008421. Participants were recruited at two sites in India: the Kala‐azar Medical Research Center (KAMRC), Muzaffarpur and Rajendra Medical Research Institute of Medical Sciences (RMRIMS), Patna; and one site in Bangladesh: Surya Kanta Kala‐azar Research Centre, International Centre for Diarrhoeal Disease Research (icddr,b). Ethical approvals were obtained from institutional ethics committees of the above centers.

Participants with PKDL were randomly allocated to the LAmB monotherapy arm or the LAmB plus miltefosine combination therapy arm. In both arms, LAmB (AmBisome®, Gilead) was given intravenously at a total dose of 20 mg/kg divided over 15 days (5 × 4 mg/kg at days 1, 4, 8, 11, and 15). In the combination therapy arm, this was combined with miltefosine (Impavido®, Knight Therapeutics), given orally twice daily for 21 days. Miltefosine was dosed allometrically for participants weighing ≤ 30 kg based on weight, height, and sex. For participants weighing > 30 kg, the allometric dose corresponded to the conventional dose of 2.5 mg/kg/day, with a maximum of 150 mg/day. Therefore, participants weighing > 30 to 44 kg received 100 mg/day and participants ≥ 45 kg received 150 mg/day.^22^ Participants were hospitalized for 15 days during LAmB administration. Miltefosine treatment began alongside LAmB and continued on an outpatient basis for a total of 21 days.

### Pharmacokinetic sampling and bioanalysis

PK assessments were conducted in a subset of 30 participants per arm. Plasma samples for LAmB were collected after the first dose (day 1) at the end of infusion, and 2, 4, 8, and 22 hours post‐infusion, and after the last dose (day 15) prior to infusion, at the end of infusion, and at 2, 6, and 22 hours post‐infusion. Additional plasma samples were collected on day 22 for the combination arm and day 30 for the monotherapy arm. Miltefosine plasma samples were collected on days 8, 15, 22, and 30 as well as at the 3‐month follow‐up visit. Skin biopsy samples were collected to measure total amphotericin B (AmB) concentrations on day 15 (monotherapy arm) and day 22 (combination arm). Skin PK data were excluded from this analysis and will be reported separately.

Plasma concentrations of total AmB and miltefosine were quantified using validated liquid chromatography coupled to tandem mass spectrometry (LC–MS/MS) assays, performed by the Lambda Therapeutic Research bioanalytical laboratory (Ahmedabad, India). Total AmB concentrations, comprising > 95% in the liposomal form,^23^ were considered the active moiety in leishmaniasis due to the preferential uptake by the MPS. Details of the AmB assay are provided in **Supplementary Methods S1**. The lower limit of quantification (LLOQ) was 500 μg/L, and assay performance met predefined acceptance criteria, with relative bias ≤ 10.3% and coefficient of variation ≤ 7.5% across tested concentrations. Miltefosine concentrations were quantified using a previously described assay (LLOQ of 4 μg/L) with minor modifications, including an upper limit of quantification (ULOQ) of 10,000 μg/L instead of 2000 μg/L.^24^

### Pharmacodynamic sampling and analysis

Clinical evaluation of PKDL lesions and parasitological examinations were performed before treatment initiation, at day 30 (end of treatment), and during follow‐up visits at 3, 6, 12, and 24 months. PKDL lesions were characterized clinically using the diagram described by Mondal et al.,^25^ where affected areas were plotted in squares on a body map. The total number of squares containing lesions was counted as the lesion score to assess disease severity. Skin snip samples were collected for parasitological assessment, and parasite load in the skin was quantified using quantitative real‐time PCR (qRT‐PCR). Measurements above the Ct cut‐off threshold were defined as 0.1 parasites/μg DNA at RMRIMS (India) and icddr,b (Bangladesh) and 0.01 parasite/μg DNA at KAMRC (India). A brief overview of the qRT‐PCR method is provided in **Supplementary Methods S2**, with further details presented in a separate article.

### Population pharmacokinetic‐pharmacodynamic analysis

Population PK‐PD analysis was performed with the nonlinear mixed‐effects modeling program NONMEM (version 7.5; ICON Development Solutions, Ellicott City, MD), aided by Perl‐speaks‐NONMEM (PsN, version 5.0)^26^ and Pirana (version 2.9.9)^27^ for run deployment. Model parameters were estimated using the first‐order conditional estimation with interaction method implemented in NONMEM. Between‐subject variability (BSV) in PK and PD parameters was estimated with an exponential variance model. For LAmB and miltefosine, proportional, additive, and combined proportional and additive error models were tested to describe the residual unexplained variability. For parasite score, an additive error model in log_10_ scale was used.

Data handling for observations below the LLOQ involved substituting the first observation below LLOQ with LLOQ/2, while subsequent observations below LLOQ were excluded. Additionally, a fixed additive residual error component of LLOQ/2 was incorporated into the model.

#### Population PK of LAmB

Liposomal drugs are known to exhibit nonlinear kinetics due to MPS phagocytosis. To account for this, a two‐compartment model with linear elimination from the central compartment and saturable distribution toward the MPS compartment was evaluated.^28^ This saturable distribution process was described by Eq. 1:

(1) $$\frac{dA_{\text{LAmB},\mathrm{MPS}}}{\mathrm{dt}}=k_{\text{in}}*A_{\text{LAmB},\text{Central}}*\left(1-\frac{A_{\text{LAmB},\mathrm{MPS}}}{B_{\max}\left(t\right)}\right)-k_{\text{out}}*A_{\text{LAmB},\mathrm{MPS}}$$

in which *B*_max_(*t*) represents the maximum drug amount the MPS compartment can take up at a given time, with *k*_in_ and *k*_out_ as the first‐order rate constants for drug uptake and efflux, respectively. Using this function, the dynamics and the extent of drug distribution are regulated by drug accumulation in the MPS compartment (*A*_LAmB,MPS_).

Following the development of the structural model, the potential effects of body weight (WT), age, geographical region (India or Bangladesh), nutritional status (informed by BMI), and treatment arm (LAmB monotherapy or combined with miltefosine) on all PK parameters were evaluated.

#### Population PK of miltefosine

The PK of miltefosine was characterized based on a previously developed population PK model in Sudanese patients with VL and PKDL.^29^ The base structural model of miltefosine applied a two‐compartment disposition with first‐order absorption and elimination to and from the central compartment. Details of model development are described in **Supplementary Methods S3**.

#### Parasite score

The qPCR level (parasites/μg DNA) in a skin snip represented parasite load in a lesion sample, while the PKDL lesion score (number of squares on the body map) described the total body areas affected by the lesions.^25^ To approximate the total *Leishmania* parasite burden for use in the PK‐PD model development, the skin qPCR level was multiplied by the PKDL lesion score, yielding a total parasite score as in Eq. 2. This calculation was based on the simplifying assumption of a homogeneous parasite load across lesions, which was considered a reasonable approximation in the absence of lesion‐specific data.

(2) $$\text{Parasite score}=\frac{\text{parasites}}{\mu g\;\mathrm{DNA}}*\text{lesion score}$$

#### Interaction between LAmB PK, the MPS, and parasite dynamics

In the absence of a specific marker for macrophage burden, it was assumed that macrophage burden associated with *Leishmania* infection partially follows the total parasite burden. Therefore, the interaction between the PK of LAmB and the MPS, indirectly informed by the parasite score, was further investigated.

Following Eq. 1, *B*_max_(*t*) represents the impact of the MPS on the distribution of LAmB over time and is further elaborated in Eq. 3:

(3) $$B_{\max}\left(t\right)=B_{\max,\text{base}}+B_{\max,\text{disease}}*A_{\text{Parasite}}\;\left(t\right)$$

where *B*_max_(*t*) is denoted as a combination of the physiological baseline accumulation of LAmB in the MPS in the absence of disease (*B*_max,base_), and the disease‐related *B*_max_ component (*B*_max,disease_), which reflects the increased macrophage burden and/or activity associated with *Leishmania* infection. In this function, *A*_parasite_(*t*) represents parasite score over time, and is characterized by a turnover model, as shown in Eq. 4:

(4) $$\frac{dA_{\text{Parasite}}}{\mathrm{dt}}=-\lambda_{\text{LAmB}}*A_{\text{LAmB},\mathrm{MPS}}*A_{\text{Parasite}}-\lambda_{\mathrm{MF}}*C_{\mathrm{MF}}*A_{\text{Parasite}}$$

Here, drug‐induced parasite clearance was modeled as a function of LAmB in the MPS compartment (*A*_LAmB,MPS_) and miltefosine concentrations in plasma (*C*_MF_), scaled by a drug‐specific factor *λ*_LAmB_ and *λ*_MF_, respectively. No synergistic or antagonistic interaction between the two drugs was assumed, based on preclinical *in vivo* studies in a murine model for VL.^30^

Parasite growth was not observed during the observation period and was likely negligible compared to drug‐induced parasite clearance. Therefore, the parasite dynamics model omitted the parasite growth rate, and the baseline parasite score was estimated. Geographical region and lesion type (e.g., macular, papules/nodules, or mixed) were evaluated as covariates on the baseline parasite score and parasite clearance.

#### Alternative LAmB dosing regimens for PKDL

The final PK‐PD model was used to simulate four treatment scenarios in a typical patient, aiming to evaluate the potential for shorter and/or lower‐dose regimens. The scenarios included (i) the trial regime of 4 mg/kg per dose, administered on days 1, 4, 8, 11, and 15 (total 20 mg/kg); (ii) a shortened duration regimen of 5 mg/kg per dose, administered on days 1, 3, 5, and 7 (total 20 mg/kg); (iii) a lower daily dose regimen of 2 mg/kg per dose, administered on days 1, 4, 8, 11, and 15 (total 10 mg/kg); and (iv) a single‐dose regimen of 10 mg/kg, as used for clinical studies in VL.^31^, ^32^

Simulations included the cumulative area under the concentration–time curve in plasma (AUC_plasma_) and the cumulative area under the amount–time curve in the MPS (AUC_MPS_), along with the corresponding parasite score over time, based on established exposure–response relationships.

#### Model evaluation

Model adequacy was guided by statistical and graphical methods as well as physiological plausibility. The change in objective function value (OFV), which equals minus twice the log‐likelihood, was used to define statistical significance between hierarchical models (chi‐square distribution with 1 degree of freedom [df]). A decrease in OFV of ≥ 3.84, representing a *P*‐value of ≤ 0.05, was considered statistically significant in this study. Individual empirical Bayes estimates were obtained using the POSTHOC option of NONMEM. R (version 4.0) and Xpose (version 4) were used for performing goodness‐of‐fit plots to assist graphical evaluation. Prediction‐corrected visual predictive checks (pcVPC) and sampling importance resampling (SIR) were performed to assess the predictive performance and the parameter precision with a 95% confidence interval (CI) for the final model.^33^, ^34^ Model simulations were performed with the mrgsolve package in R.

## RESULTS

### Participants and sampling

In total, 60 participants were included in this PK‐PD analysis: 10 from Bangladesh and 50 from India. Within each site, participants were equally allocated to the two treatment arms, LAmB monotherapy (*n* = 30) and LAmB plus miltefosine combination therapy (*n* = 30). Demographics and baseline characteristics are summarized in **Table** **1**.

**Table 1:** Patient demographics

Parameter	Overall (n=60)
No. of patients per country, (*n*, %)	
Bangladesh	10 (16.7%)
India	50 (83.3%)
Treatment arm (*n*, %)	
Liposomal amphotericin B	30 (50.0%)
Liposomal amphotericin B + Miltefosine	30 (50.0%)
Sex (*n*, %)	
Female	32 (53.3%)
Male	28 (46.7%)
Age (years), median [IQR]	22.5 [15.8, 36.3]
Age group (*n*, %)	
Adult (> 18 years)	42 (70.0%)
Pediatrics (≤ 18 years)	18 (30.0%)
Body weight (kg), median [IQR]	45.5 [40.3, 55]
Fat free mass (kg), median [IQR]	39.2 [34.1, 45]
Height (cm), median [IQR]	155 [149, 162]
Body mass index (kg/m^2^), median [IQR]	19.0 [17.5, 20.6]
Baseline skin parasite qPCR (parasite/μg DNA in a skin snip), median [IQR]	1050 [156, 5920]
Baseline PKDL lesion score (no. of squares on the manikin), median [IQR]^25^	175 [75.5, 262]

**Figure 1:**
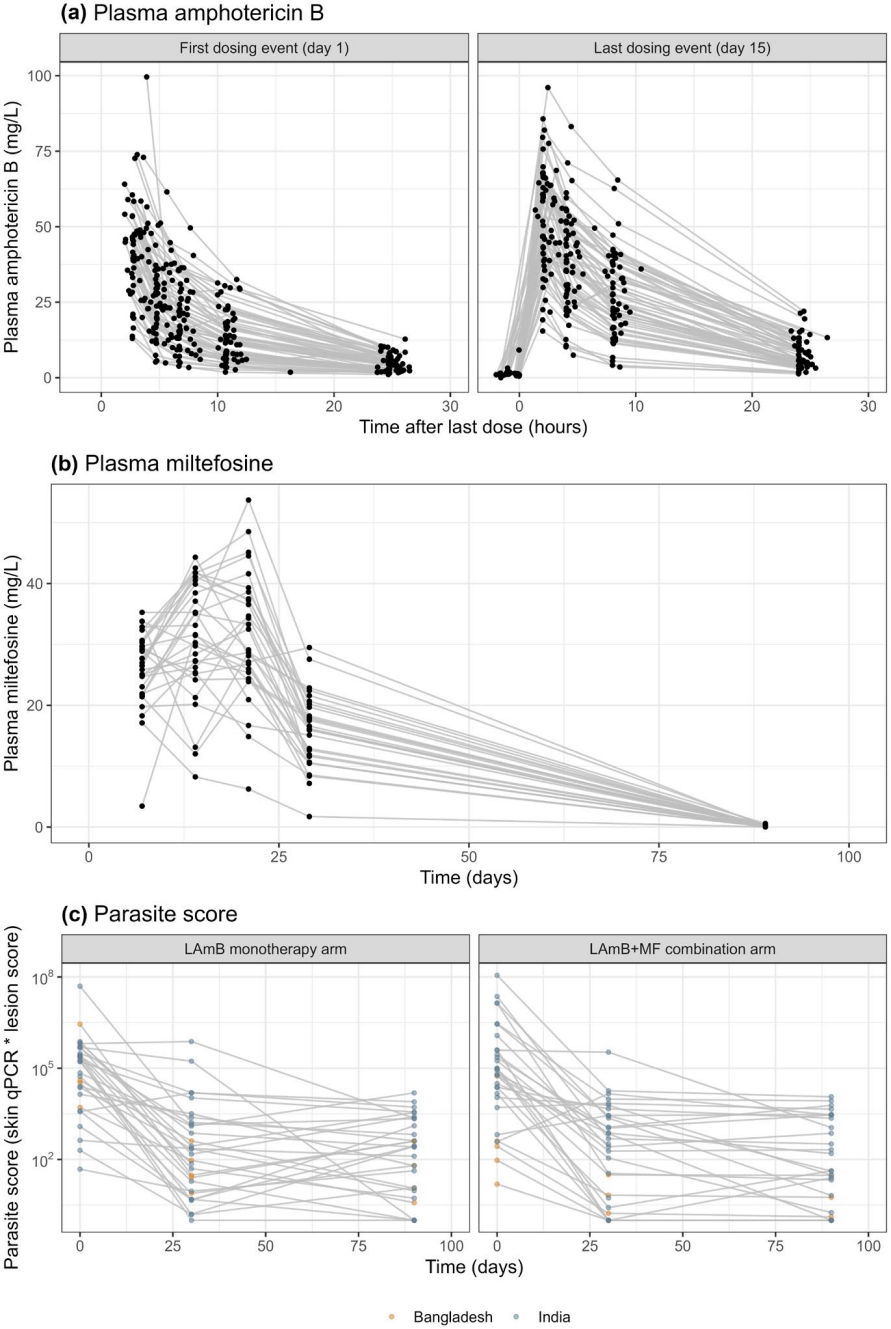
Observation‐time profiles of (**a**) plasma total amphotericin B concentration‐time profile, (**b**) plasma miltefosine concentration‐time profile, and (**c**) parasite score up to the 3‐month follow‐up visit. The parasite score was informed by multiplying skin qPCR value (parasites/μgDNA in a skin snip) by PKDL lesion score (squares on the body map). Blue dots represent data from India and yellow dots represent data from Bangladesh.

The PK analysis included 646 AmB and 143 miltefosine plasma samples, with 66 AmB samples below the LLOQ, which were fixed to LLOQ/2 (0.25 mg/L). The PD analysis incorporated skin qPCR and PKDL lesion score assessed at baseline, day 30, and 3‐month follow‐up visit. For the 34 unquantifiable skin qPCR measurements, values were inputted as 0.1 parasites/μg DNA (RMRIMS and icddr,b) or 0.01 parasites/μg DNA (KAMRC). In total, 177 parasite score records were included in the PK‐PD model. **Figure** **1** presents the observed plasma concentrations of total AmB and miltefosine over time, and parasite scores from baseline to the 3‐month follow‐up.

### Population PK‐PD analysis

**Figure 2:**
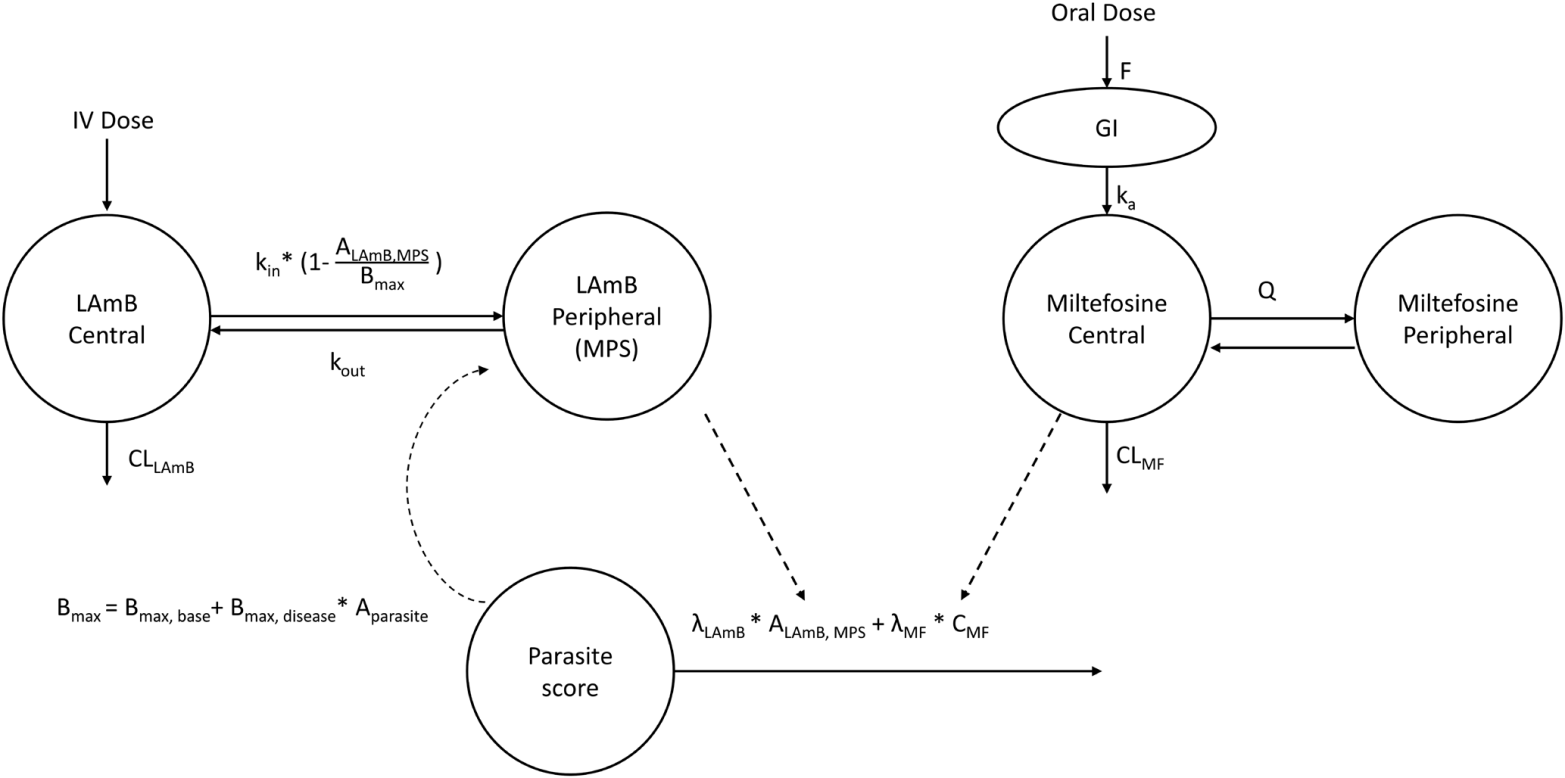
Schematic representation of the PK‐PD model linking LAmB, miltefosine, and parasite dynamics. LAmB disposition was described by a central and MPS compartment with nonlinear uptake limited by *B*_max_, which decreases as parasite burden declines. Miltefosine was modeled with oral absorption, central and peripheral compartments, and linear clearance. Parasite clearance was driven by LAmB in the MPS and miltefosine plasma concentrations, with parasite reduction feeding back to increase systemic LAmB exposure. Abbreviations: *A*_LAmB_,_MPS_, LAmB amount in MPS compartment; *A*_parasite_, parasite score; *B*_max_, the maximal drug accumulation in the peripheral MPS compartment; *B*_max,base_, physiological baseline *B*_max_; *B*_max,disease_, disease‐related effect on B_max_; CL, drug clearance from the central compartment; *F*, relative bioavailability; GI, gastrointestinal tract; *k*_a_, first‐order absorption rate; *k*_in_, first‐order rate constant of distribution to MPS compartment; *k*_out_, first‐order rate constant of release from MPS compartment; LAmB, liposomal amphotericin B; MPS, mononuclear phagocytic system; *Q*, intercompartmental clearance; *λ*_LAmB_, coefficient of LAmB amount in the MPS on parasite clearance; *λ*_MF_, coefficient of miltefosine plasma concentration on parasite clearance.

The structure of the final PK‐PD model is depicted in **Figure** **2**, while the parameter estimates and their precisions are summarized in **Table** **2**.

**TABLE 2:** Final parameter estimates

Pharmacokinetics of liposomal amphotericin B (LAmB)			
Parameter	Unit	Estimate (SIR 95% CI)	BSV in CV% (SIR 95% CI)
CL_LAmB_ a	L/h	0.3 (0.26–0.33)	43 (37–51)^b^
Vc_LAmB_ a	L	3.1 (2.8–3.4)	32 (26–40)^b^
*k*_in_	1/h	0.53 (0.44–0.65)	59 (38–78)
*k*_out_	1/h	0.018 (0.014–0.024)	–
*B*_max,base_ a	mg	10 (fixed)	–
*B*_max,disease_	mg/log_10_(parasite score)	16 (15–18)	40 (30–51)
Residual variability			
Proportional error in LAmB	CV%	22 (21–24)	–
Additive error in LAmB	mg/L	0.25 (fixed)	–

Pharmacokinetics of miltefosine (MF)			
Parameter	Unit	Estimate (SIR 95% CI)	BSV in CV% (SIR 95% CI)
CL/F_MF_ c	L/h	0.086 (0.079–0.095)	19 (14–25)
Vc/F_MF_ c	L	20 (18–22)	‐
*Q*/F_MF_	L/h	0.0012 (0.0009–0.0017)	‐
Vp/F_MF_ c	L	1.9 (1.3–3.3)	‐
*k*_a_	1/h	0.225 (fixed)^d^	‐
COV_F,CumDose_	Exponent	−0.13 (fixed)^d^	‐
Residual variability			
Proportional error in MF	CV%	27 (23–31)	‐

Pharmacodynamics of LAmB and MF on parasite score			
Parameter	Unit	Estimate (SIR 95% CI)	BSV in CV% (SIR 95% CI)
*λ*_LAmB_	1/mg h	1.6 × 10^−4^ (1.3 × 10^−4^–2.1 × 10^−4^)	49 (28–64)
*λ*_MF_	L/mg h	4.9 × 10^−5^ (1.3 × 10^−5^–8.2 × 10^−5^)	–
Baseline parasite score	log_10_ (parasite score)	4.7 (4.4–5.1)	350 (185–316)
Residual variability			
Additive error in parasite score	log_10_ (parasite score)	1.1 (0.89–1.3)	–

*λ*_LAmB_, coefficient of AmB amount in MPS on parasite clearance rate; *λ*_MF_, coefficient of miltefosine plasma concentration on parasite clearance rate; BSV, between‐subject variability; SIR, sampling importance resampling; *B*_max_, the maximal drug accumulation in the peripheral MPS compartment; *B*_max,base_, physiological baseline *B*_max_; *B*_max,disease_, disease‐related effect on *B*_max_; CL, drug clearance from the central compartment; COV_F,CumDose_, exponent of power relationship between cumulative MF dose and bioavailability; *k*_a_, first‐order absorption rate; *k*_in_, first‐order rate constant of distribution to MPS compartment; *k*_out_, first‐order rate constant of release from MPS compartment; *Q*, intercompartmental clearance; Vc, volume of distribution of the central compartment; Vp, volume of distribution of the peripheral compartment.

^a^ Allometrically scaled based on body weight with a power exponent of 0.75 for clearance and 1 for the volume of distribution and B_max,base_. The estimate is given for a standardized body weight of 45 kg.

^b^ Correlation between BSV in CL_LAmB_ and Vc_LAmB_ is estimated at 92% (SIR 95% CI: 61–100).

^c^ Allometric scaled based on fat‐free mass with a power exponent of 0.75 for clearance and 1 for volume of distribution. Estimate is given for a standardized fat‐free mass of 40 kg.

^d^ Parameter fixed to the value identified in the previous study based on Sudanese PKDL patients.^29^

#### Structural model of LAmB and miltefosine

The LAmB plasma concentration‐time profiles observed (**Figure** **1** **a**) indicated potential nonlinear kinetics. Notably, LAmB plasma AUC_0–24h_ was generally higher at the last dosing event compared to the first, whereas the trough concentration ratio between these events indicated no evidence of drug accumulation. The data was best described by a two‐compartment model with a saturable distribution process (Eq. 1), consistent with the primary distribution pathway of liposomal drugs through MPS‐mediated phagocytosis.^9^, ^10^

The previously developed PK model structure for miltefosine in Sudanese patients with PKDL adequately described the PK in the ISC cohort (details in **Supplementary Results S1**).^29^ In the final model, CL/F_MF_, Vc/F_MF_, and Vp/F_MF_ were estimated at 0.086 L/h (95% CI: 0.079–0.095), 20 L (95% CI: 18–22), and 1.9 L (95% CI: 1.3–3.3), respectively, normalized to a median fat‐free mass (FFM) of 40 kg. An effect of cumulative miltefosine dose on oral bioavailability was identified, with the exponent fixed at −0.13.

#### Interaction between LAmB, the MPS, and parasite dynamics

Parasite dynamics were modeled up to the 3‐month follow‐up visit, corresponding to the last PK observation. Most patients showed a more than 2 log_10_ reduction in parasite score following either monotherapy or combination therapy (**Figure** **1** **c**). The factors *λ*_LAmB_ and *λ*_MF_ for drug‐induced parasite clearance were estimated at 1.6 × 10^−4^ L/mg h (95%: 1.3 × 10^−4^ – 2.1 × 10^−4^) and 4.9 × 10^−5^ L/mg h (95% CI: 1.3 × 10^−5^ – 8.2 × 10^−5^), respectively. During the model building process, LAmB concentration and AUC until the last observation in the central compartment were also evaluated as predictors for drug‐induced parasite clearance. Notably, the former led only to a marginal improvement in model fit, while the latter worsened the model fit.

In the final PK‐PD model, WT was incorporated as a covariate on CL_LAmB_, *V*c_LAmB_ and *B*_max,base_ using an allometric scaling approach with a fixed exponent of 0.75, 1 and 1, respectively; estimation of these exponents did not improve the fit of the model. These parameters were normalized to the median WT of 45 kg, estimated at 0.30 L/h (95% CI: 0.26–0.33) for CL_LAmB_ and 3.1 L (95% CI: 2.8–3.4) for *V*c_LAmB_. *B*_max,base_ was first estimated and later fixed to 10 mg to improve model stability. The value of *B*_max,disease_ was 16 mg/log_10_(parasite score) (95% CI: 15–18), indicating a positive correlation between total parasite and the capacity for LAmB accumulation in the MPS compartment. The rate constants *k*_in_ and *k*_out_ to the MPS compartment were estimated at 0.53 1/h (95% CI: 0.44–0.65) and 0.018 1/h (95% CI: 0.014–0.024), respectively.

No significant effects of age, geographical region, or nutritional status on any PK parameters were identified. Similarly, no significant differences in CL_LAmB_, Vc_LAmB_, or *k*_in_ were detected between the combination and monotherapy arms, suggesting no relevant miltefosine interaction on the PK of LAmB. Although including geographical region as a covariate on baseline parasite score, with lower values in Bangladesh compared to India, significantly improved the OFV, it was excluded from the final model due to model instability and low parameter precision. Additionally, no significant differences in parasite clearance were found between the geographical regions or lesion types. Overall, the final model adequately described the plasma concentrations of LAmB and miltefosine, as well as parasite scores. Model evaluations (**Supplementary Results S2**) showed no notable trends or biases in predictions based on goodness‐of‐fit plots, and the simulations adequately captured observations in the pcVPC.

#### Link between parasite dynamics and increased LAmB systemic exposure

**Figure 3:**
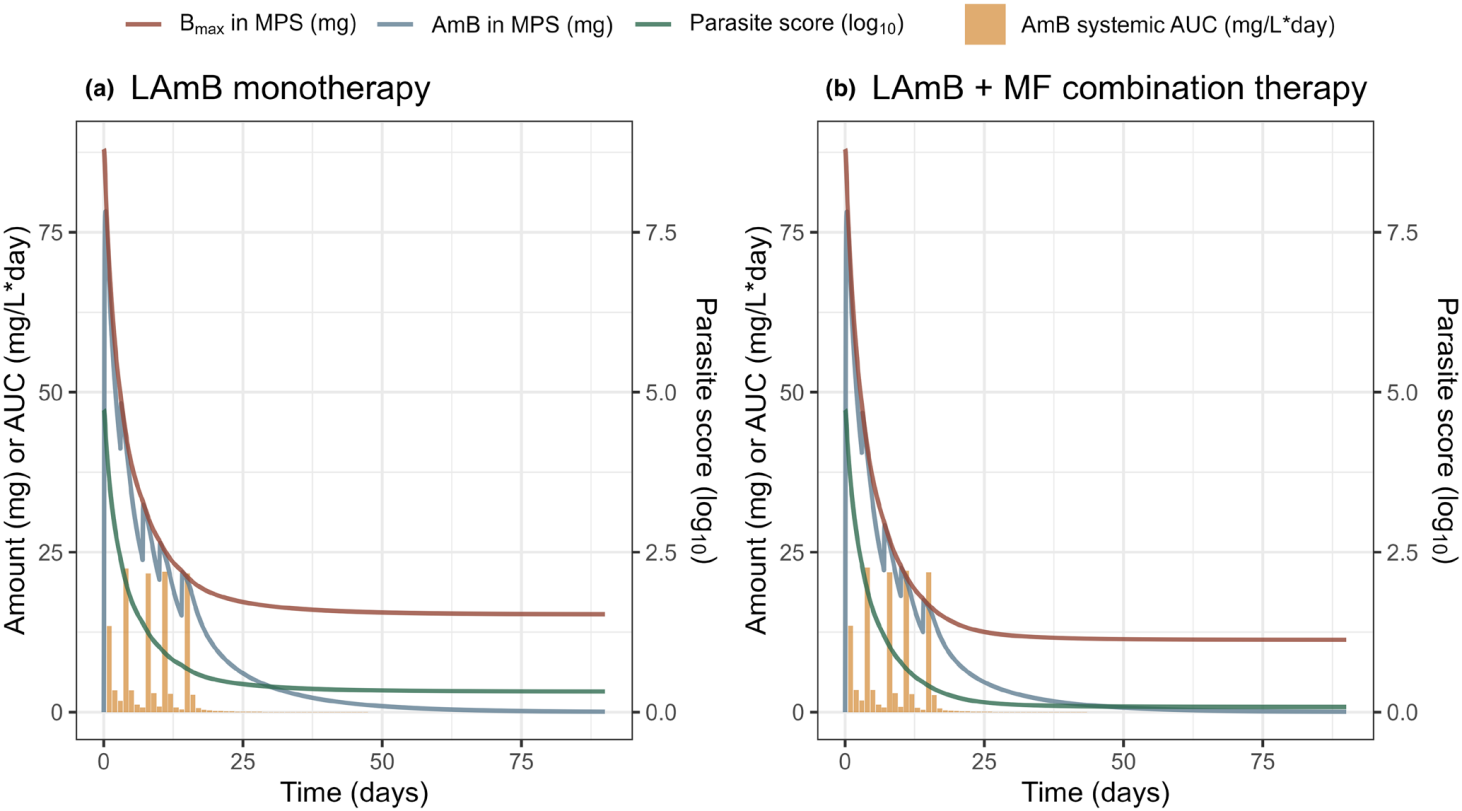
Simulations in a typical patient receiving (**a**) LAmB monotherapy or (**b**) LAmB and miltefosine (MF) combination therapy. The typical patient has body weight of 45 kg, fat‐free mass of 40 kg, and baseline parasite score of 10^4.7^. A higher *B*_max_ (red line) at treatment start results in more AmB uptake by the MPS (blue line) and subsequently lower daily AUC of systemic AmB (yellow bars). The combination treatment results in a greater decrease in parasite score (green line) and subsequently a more pronounced decrease in *B*_max_ (red line).

To elucidate the interplay between the PK of LAmB, the MPS, and parasite dynamics, simulations were performed based on a typical patient with WT of 45 kg, FFM of 40 kg, and a log_10_ baseline parasite score of 4.7, receiving either LAmB monotherapy or in combination with miltefosine. As illustrated in **Figure** **3**, the combination of miltefosine with LAmB resulted in a more than 6‐fold greater reduction in parasite score by the 3‐month follow‐up visit, compared to treatment with LAmB alone. Importantly, this parasite reduction was accompanied by a 22% decrease in B_max_ by day 15, which translated into a 54% increase in systemic LAmB exposure (AUC_0‐24h_) compared with day 1. These results demonstrate that declining parasite burden can directly increase systemic LAmB exposure.

#### Alternative LAmB dosing regimen simulations

**Figure 4:**
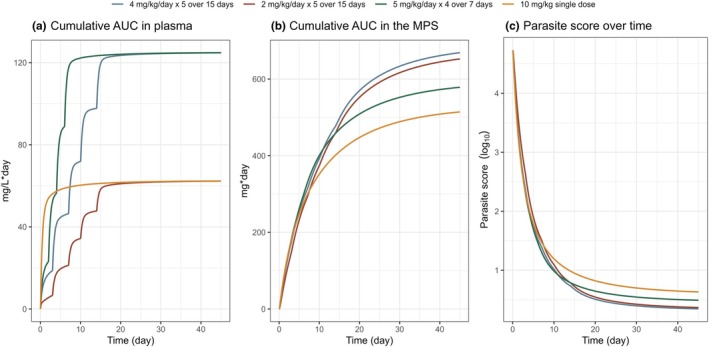
Effect of alternative LAmB dosing strategies on (**a**) cumulative area under the concentration–time curve in plasma, (**b**) cumulative area under the amount–time curve in the MPS, and (**c**) parasite score over time. Four regimens were simulated: trial regimen (4 mg/kg on days 1, 4, 8, 11, 15; blue), lower daily dose (2 mg/kg on days 1, 4, 8, 11, 15; red), shortened regimen (5 mg/kg on days 1, 3, 5, 7; green), and single dose (10 mg/kg; yellow). Reducing the total LAmB dose to 10 mg/kg with the lower daily dose regimen (red) resulted in a similar cumulative area under the amount–time curve in the MPS and parasite score reduction as the trial regimen (blue), despite a lower cumulative area under the concentration–time curve in plasma, and outperformed the same total dose given as a single 10 mg/kg administration (yellow). These results suggest that lower daily doses may be considered for PKDL while maintaining the same dosing interval.

Model simulations (**Figure** **4**) indicated that administering lower daily LAmB doses over a longer treatment duration resulted in increased LAmB accumulation in the MPS compartment and subsequently improved parasite response. For example, a total dose of 10 mg/kg, divided into five doses of 2 mg/kg per administration given over 15 days, achieved a lower parasite score during the follow‐up period compared to a 10 mg/kg single‐dose regimen. The difference in parasite clearance was attributed to comparable saturation levels within the MPS compartment achieved with the doses ranging from 2 to 10 mg/kg per administration.

In this clinical study, participants received a total LAmB dose of 20 mg/kg, divided into five administrations over 15 days. Reducing the total LAmB dose to 10 mg/kg through lower daily doses resulted in a similar AUC_MPS_, achieving a comparable reduction in parasite score despite a lower AUC_plasma_. These findings suggest that a lower daily dose could be considered for PKDL while maintaining the same dosing interval.

## DISCUSSION

This study presents the first semi‐mechanistic PK‐PD model of LAmB in patients with PKDL, revealing a complex bidirectional interaction between drug disposition and parasite burden. For the first time, the LAmB PK was characterized in leishmaniasis patients, showing saturable uptake into the MPS, most likely reflecting phagocytosis by macrophages. Given the large increase in macrophages associated with leishmaniasis pathogenesis, the MPS uptake capacity for LAmB was assumed to correlate with parasite score. As treatment reduced parasite burden, the MPS uptake capacity decreased, resulting in increased systemic LAmB exposure during the treatment period.

Model‐based simulations (**Figure** **4**) indicated that reducing the daily LAmB dose from 4 mg/kg to 2 mg/kg, while maintaining five administrations over 15 days, was sufficient to saturate the MPS at each administration. This lower dose yielded comparable MPS exposure and parasite clearance, suggesting the total dose could be halved without compromising efficacy. Such a dose reduction may improve safety, lower costs, and enhance programmatic feasibility in endemic regions. These findings are consistent with previous evidence: in VL, multiple low‐dose regimens achieved faster parasite clearance than single high‐dose regimens,^32^ and a PKDL study in Bangladesh demonstrated satisfactory results with a total LAmB dose of 15 mg/kg divided over 2 weeks.^35^ Together with our results, these observations strengthen the rationale for LAmB dose‐reduction strategies in PKDL.

To mechanistically describe the saturable distribution of LAmB, the model incorporated a time‐varying maximal uptake capacity, *B*_max_(*t*), comprising both a baseline physiological component and a disease‐driven component that scaled with parasite score. Distribution into the peripheral MPS compartment was estimated to be five times faster than systemic elimination, underscoring the dominant role of macrophages in drug disposition.^21^ In most patients, MPS concentrations plateaued within 2–4 hours after infusion, followed by a slow distribution back into the systemic circulation. By the last administration on day 15, the remaining MPS uptake capacity was less than 50% of baseline, explaining the higher systemic drug exposure after repeated dosing.

In all forms of leishmaniasis, macrophage activation plays an important role in disease progression and recovery.^19^, ^20^ Therefore, liposomal formulations such as LAmB exploit this biology by targeting the MPS, where parasites primarily reside. Supported by the model, drug concentrations in the MPS, rather than plasma, were the main driver of parasite clearance, indicating that systemic exposure is not an adequate surrogate for treatment response. Notably, by day 15, *B*_max_(t) had declined by 22% under combination therapy, resulting in a 54% increase in systemic AUC_0–24h_ compared with day 1. These findings demonstrate that infection‐driven and treatment‐driven changes in LAmB PK restrict the predictive value of plasma concentrations alone for treatment efficacy. Therefore, systemic exposure should not be used as the sole target for optimizing future treatment strategies.

An important rationale for treating PKDL is to prevent the transmission of *Leishmania* parasites. The combination of miltefosine with LAmB led to a greater reduction in parasite score compared to treatment with LAmB alone (**Figure** **3**). This result underscores the potential of combination therapy not only to enhance individual treatment outcomes but also to offer a promising approach for controlling disease transmission.

Care should be taken when extrapolating these results to other populations or clinical presentations of leishmaniasis, where parasites and associated macrophage burdens might affect LAmB disposition differently. Moreover, the suggested dosing reductions require validation in prospective clinical trials before informing treatment guidelines. Parasite score data were sparse during treatment and were derived under the simplified assumption of a homogeneous parasite load across lesions, which may limit detailed characterization of PK‐PD dynamics. Additionally, since there were no patients who received miltefosine alone, the “true” miltefosine‐induced parasite clearance could not be estimated separately.

In summary, this study provides new mechanistic insight into the relationship between host macrophages, parasite burden, and LAmB pharmacology in PKDL. It demonstrates that drug levels within the MPS, not plasma, drive parasite clearance, challenging conventional reliance on systemic exposure as a marker for efficacy. Model‐based simulations suggest that lower daily doses of LAmB, administered with the same schedule, may lead to similar parasite clearance and efficacy, while improving safety, affordability, and programmatic feasibility. If confirmed in clinical trials, these strategies could contribute to more rational and cost‐effective treatment approaches for PKDL and other forms of dermal leishmaniasis.

## FUNDING

The Drugs for Neglected Diseases initiative (DNDi) is grateful to its donors, public and private, who have provided funding to DNDi since its inception in 2003. A full list of DNDi’s donors can be found at http://www.dndi.org/about/donors/. TD was supported by the Dutch Research Council (NWO/ZonMw; project 91617140) and the Swedish Research Council (VR 2022‐01251).

## CONFLICT OF INTEREST

The authors declared no competing interests for this work.

## AUTHOR CONTRIBUTIONS

All authors wrote the manuscript; S.S., O.P.S., D.M., K.P., P.D., F.A., and T.P.C.D. designed the research; W.‐Y.C., O.P.S., S.S., D.M., K.P., P.D., S.R., A.T., E.C, A.D.R.H, F.A, and T.P.C.D performed the research; W.‐Y.C., A.D.R.H., and T.P.C.D. analyzed the data; I.R., A.T., and E.C. contributed new reagents/analytical tools.

## Supporting information


Data S1
