# Supplementary material for: Bidirectional Interaction Between Liposomal Amphotericin B Pharmacokinetics and Parasite Dynamics in Patients With Post‐Kala‐Azar Dermal Leishmaniasis: Potential Implications for Optimal Dosing
Source: Clin Pharmacol Ther. 2025 Nov 25;119(2):437–46. doi: 10.1002/cpt.70124 (PMC12816433; doi:10.1002/cpt.70124)
Supplement: Supplementary file 1 — Data S1 [file CPT-119-437-s001.pdf]

Supplementary file

Contents

Supplementary Methods S1. Bioanalytical assay for amphotericin B in human plasma -------------- 2

Supplementary Methods S2. Quantitative real-time PCR methods --------------------------------------- 3

Supplementary Methods S3. Miltefosine PK model development -- 4

Supplementary Results S1. Miltefosine PK and covariate analysis -- 5

Supplementary Results S2. Model evaluations

Supplementary Figure S1. Goodness-of-fit of the final PK-PD model ---------------------------------- 6

Supplementary Figure S2. Prediction-corrected visual predictive checks ----------------------------- 9

Supplementary Methods S1. Bioanalytical assay for amphotericin B in human plasma

A validated LC-MS/MS assay was employed. Calibration standards and quality control (QC) samples were prepared in human K2EDTA plasma, with a validated calibration range of 0.500–100 µg/mL. Samples were stored at -65°C prior to analysis. For sample preparation, 50 µL of plasma was mixed with 100 µL of internal standard solution (natamycin). Protein precipitation was performed by adding 750 µL of 2 mM ammonium formate in water (pH 3.0)/methanol mixture (2:8, v/v) to each sample. The mixture was centrifuged at 14,000 x g for 10 min at ≤10°C. The resulting supernatant was injected into the LC-MS/MS system for analysis. Chromatographic separation was performed using an Acquity UPLC system (Waters Corporation, Milford, MA, USA) coupled to a TSQ Quantum Ultra triple quadrupole mass spectrometer (Thermo Fisher Scientific, Waltham, MA, USA). An Eclipse XDB-C8 column (150 × 4.6 mm, 5 µm particle size; Agilent Technologies, Santa Clara, CA, USA) was used for separation. The mobile phase consisted of 2 mM ammonium formate in water (pH 3.0; mobile phase A) and methanol (mobile phase B). Chromatographic separation was achieved using isocratic elution with 75% mobile phase B at a flow rate of 0.7 mL/min, resulting in a total run time of 4.2 min. Detection was performed in positive electrospray ionization mode using selected reaction monitoring (SRM). The mass-to-charge (m/z) transitions monitored were 924.4 → 743.3 for amphotericin B and 666.2 → 503.3 for the internal standard, natamycin. Assay performance met acceptance criteria, with a relative bias of ±10.3% at the LLOQ and ±9.0% at concentrations above the LLOQ. The coefficient of variation (CV) was ≤6.4% at the LLOQ and ≤7.5% at higher concentrations.

Supplementary Methods S2. Quantitative real-time PCR methods

DNA was isolated from skin snips using a QIA amp DNA tissue & blood mini kit (Qiagen, Hilden, Germany) as per the manufacturer's instructions and stored at -20˚C until quantitative real-time PCR (qRT-PCR). DNA concentration and quality were assessed by UV7Vis spectrophotometer measurements (ND-2000 spectrophotometer from Thermo Scientific, MA, USA; UV/Vis Nano spectrophotometer from Nabi, South Korea). Parasitological assessments were done by using qPCR based on the amplification of kinetoplastid DNA (kDNA) (at KAMRC and RMRIMS) or small subunit ribosomal ribonucleic acid (SSU rRNA). Every qRT-PCR run had a standard curve performed with serially diluted 10-fold DNA from *Leishmania donovani* (L.donovani MHOM/IN/80/DD8). The parasite concentration was divided by the DNA concentration of the snip to provide values for parasites/µg. The normality of distributions was checked using the Shapiro-Wilk test. Accordingly, the Mann-Whitney U test was used to analyse differences between the different groups. Significance was set at $p<0.050$. All calculations were made using GraphPad Prism v. 8 software. Table S1 summarizes the qPCR methods employed at each site.

Table S1. qPCR measurement methods

	**KAMRC (India)**	**RMRIMS (India)**	**ICDDRB (Bangladesh)**
Chemistry	Taqman chemistry	SYBR Green I	Taqman chemistry
Forward primer	5'-CTTTTCTGGTCCTCCGGGTAGG-3'	5'-CTTTTCTGGTCCTCCGGGTAGG-3'	5'-GGTTCCTTTCCTGATTTACG-3'
Reverse primer	5'-CCACCCGGCCCTATTTTACACCAA-3	5'-CCACCCGGCCCTATTTTACACCAA-3	5'-GGCCGGTAAAGGCCGAATAG-3'
Master mix	2X Taqman Universal PCR master mix (Applied Biosystems)	2X SYBR green master mixture (Roche)	2X Taqman Universal PCR master mix (Applied Biosystems)
Real-Time PCR System	ABI 7500 (Applied Biosystem)	LightCycler480 Real-Time PCR system (Roche)	Biorad CFX-96 (Biorad)
Target	Kinetoplast DNA	Kinetoplast DNA	SSU-rRNA
Median slope (IQR)	-3.50 (3.53 – 3.24)	-3.51 (3.60 - 3.16)	-3.42 (3.58 - 3.28)
Median efficiency (IQR) %	93.05 (91.78 – 103.30)	92.51 (89.30- 106.87)	95.84 (89.99- 101.52)
Reference	Verma et al. [1]	Verma et al.[1]	Hossain et al. [2]

Supplementary Methods S3. Miltefosine PK model development

The PK of miltefosine was characterized based on a previously developed population PK model in Sudanese patients with VL and PKDL.[3] The base structural model of miltefosine applied a two-compartment disposition with first-order absorption and elimination to and from the central compartment. To account for body size variations, fat-free mass (FFM) was employed as a descriptor, and its relationship with CL/F_{MF} and Vd/F_{MF} was determined using allometric scaling with power exponents of 0.75 and 1, respectively. Since only sparse miltefosine PK observations were available from one week after treatment start, the absorption rate constant (k_a) was fixed to 0.225 1/h according to the results of Sudanese PKDL patients.[3]

In the previous PK study, a significant decrease in miltefosine relative oral bioavailability (F) during the first week of treatment was found in VL patients, indicating a temporary malabsorption associated with VL disease in Eastern Africa.[3] In addition, in both VL and PKDL patients, a decrease in F was found with cumulative dose of miltefosine, suggesting saturation in membrane translocation of miltefosine in the gut.[3] These two effects were therefore evaluated as described in Equation S1.

(Equation S1)

$$F\ (if\ treatment\ day\ \leq 7\ ) = TVF\ * \left(1 - COV_{F,W1}\right) * CumDose\ \left(\frac{mg}{kg}\right)^{COV_{F,CumDose}}$$

$$F\ (if\ treatment\ day >\ 7\ ) = TVF\ \ * CumDose\ \left(\frac{mg}{kg}\right)^{COV_{F,CumDose}}$$

In this function, TVF represents typical value of F, which was fixed to 1. $COV_{F,\ W1}$ represents the covariate effect of disease-related malabsorption on the reduced F during the first week of treatment. CumDose represents cumulative miltefosine dose (mg/kg), derived from the sum of weight normalized daily dose. $COV_{F,\ CumDose}$ represents the continuous covariate effect of cumulative miltefosine dose on F over the 21-day treatment, described with a power function.

Supplementary Results S1. Miltefosine PK and covariate analysis

The previously developed PK model for miltefosine in Sudanese PKDL patients adequately described the PK profiles in the ISC cohort.[3] A two-compartment disposition model with first-order absorption and elimination from the central compartment best described the data. BSV was identifiable only in CL_{MF}/F, with an estimated 19% CV (95% CI: 14–25). Similar to Sudanese patients,[3] no decrease in relative F during the first treatment week was observed. An effect of cumulative miltefosine dose on F was supported but imprecisely estimated; therefore, the cumulative dose effect ($COV_{F,CumDose}$) was fixed at -0.13, consistent with the Sudanese study, resulting in a 12-unit drop in OFV.

In the final model, CL/F_{MF}, Vc/F_{MF} and Vp/F_{MF} were estimated at 0.086 L/h (95%CI: 0.079-0.095), 20 L (95%CI: 18-22), and 1.9 L (95%CI: 1.3-3.3), respectively, normalized to a median FFM of 40 kg. The final model adequately described miltefosine concentrations, with GOF plots and VPC (*Supplementary Results S2*) showing no major deviations.

Supplementary Results S2. Model evaluations

Supplementary Figure S1. Goodness-of-fit of the final PK-PD model

(A) Liposomal AmB PK model. The black dots represent patients from the LAmB monotherapy arm and the gray dots represent patients from the LAmB+miltefosine combination therapy arm.

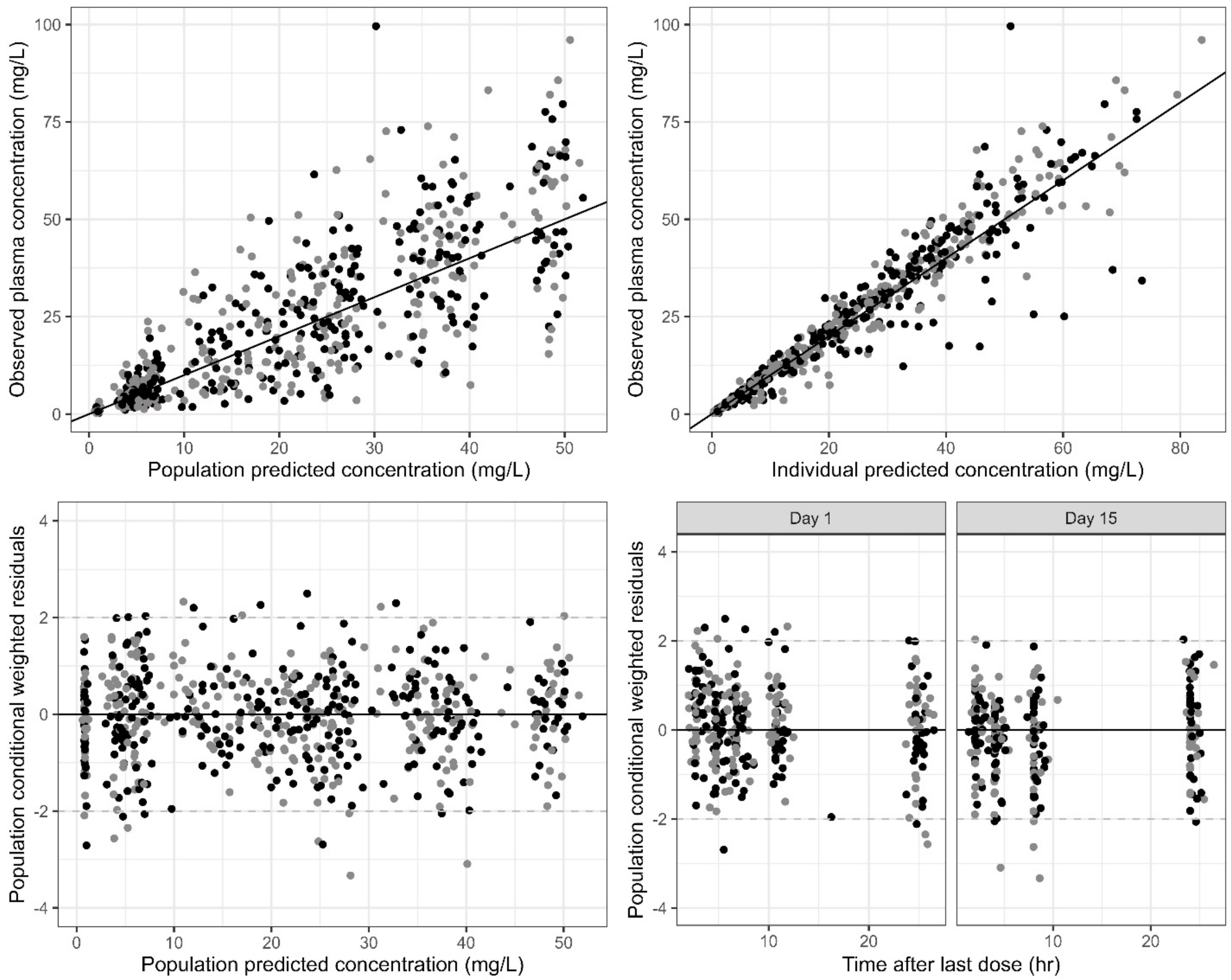

(B) Miltefosine PK model

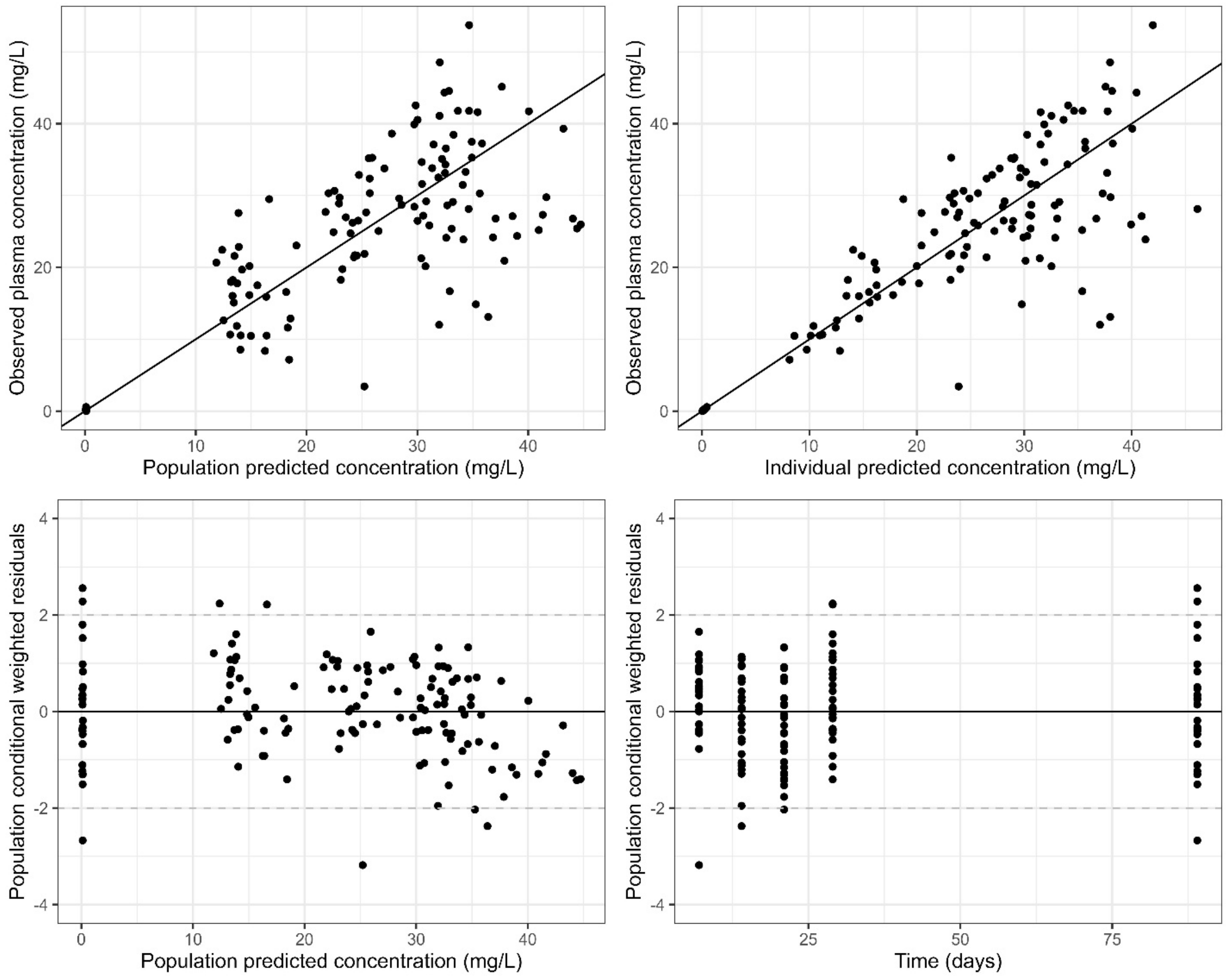

(C) Skin parasite score model. The black dots represent patients from the LAmB monotherapy arm and the gray dots represent patients from the LAmB+miltefosine combination therapy arm.

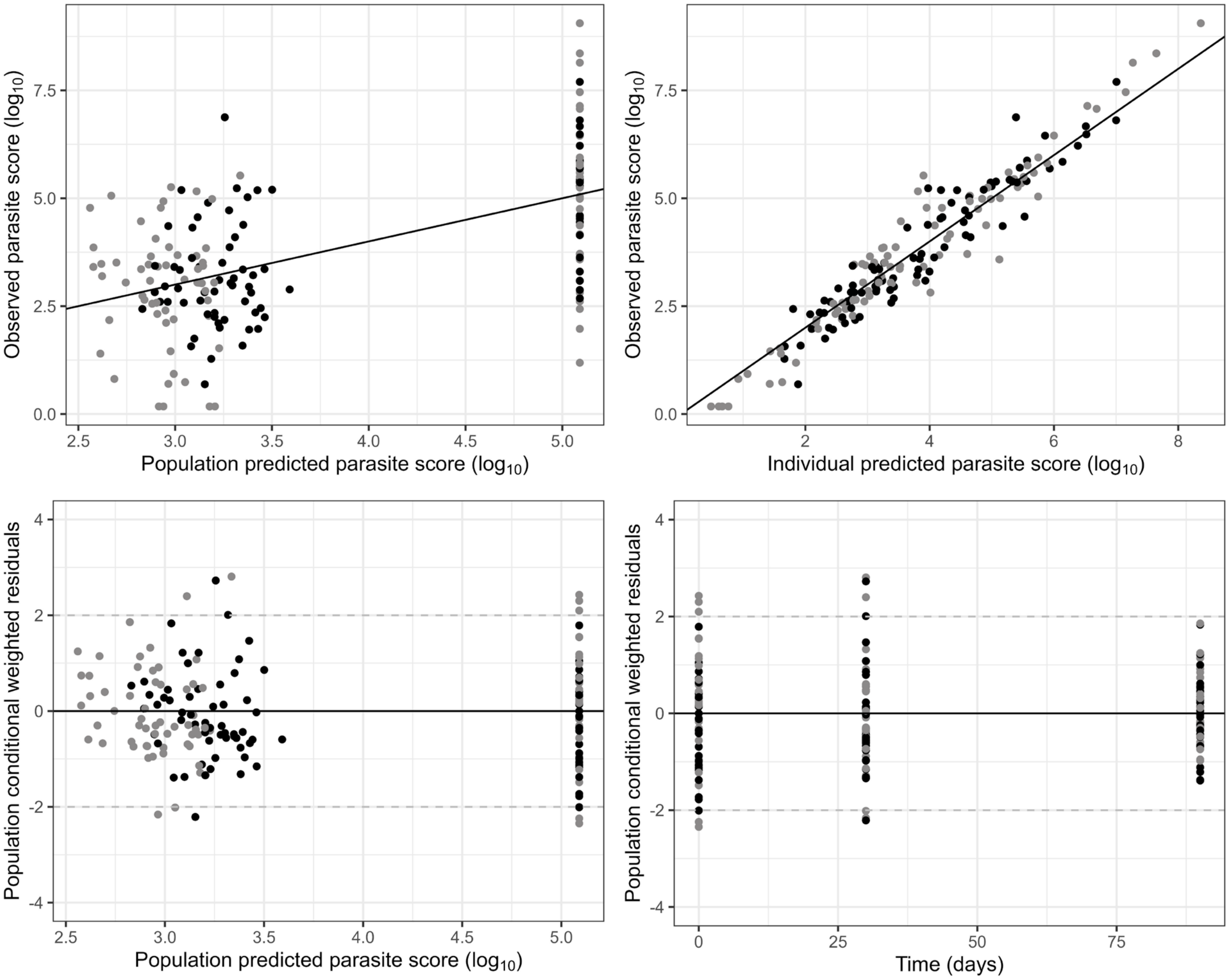

Supplementary Figure S2. Prediction-corrected visual predictive checks for (A) the PK model of liposomal AmB (B) the PK model of miltefosine (C) the PD model of skin parasite score.

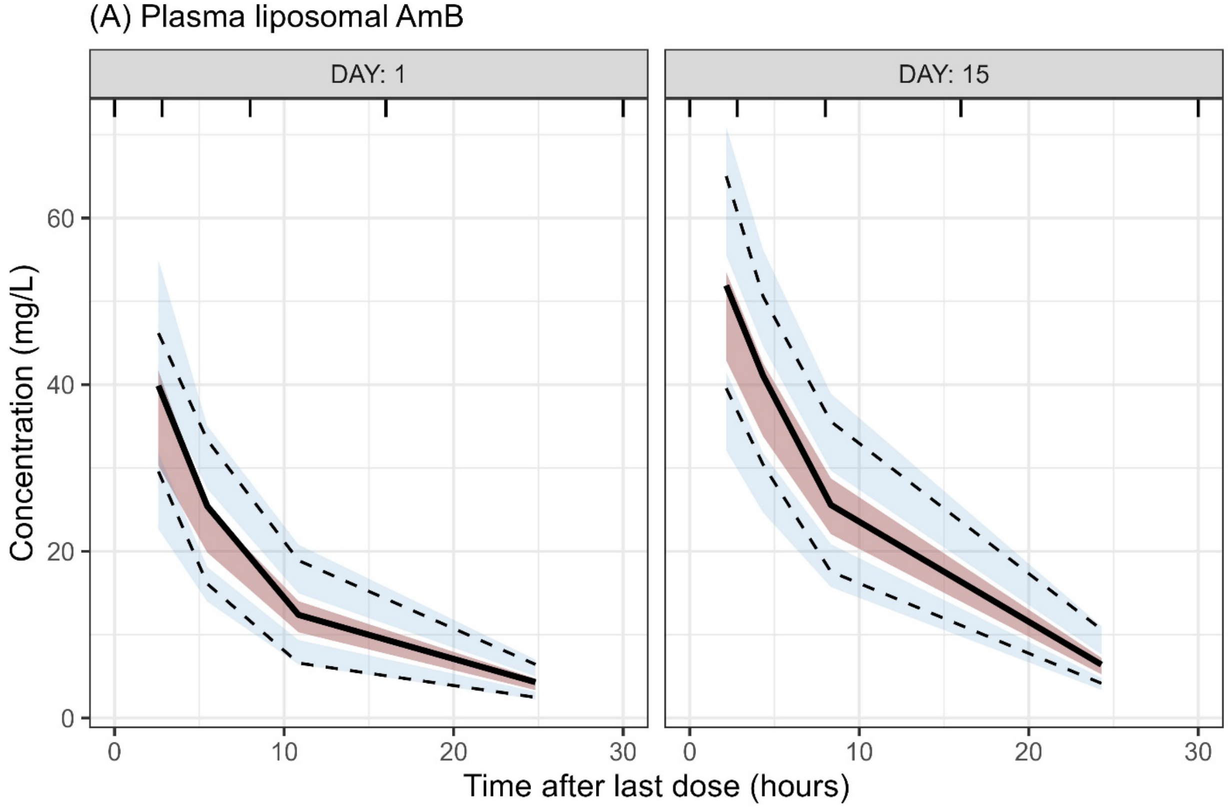

(B) Plasma miltefosine

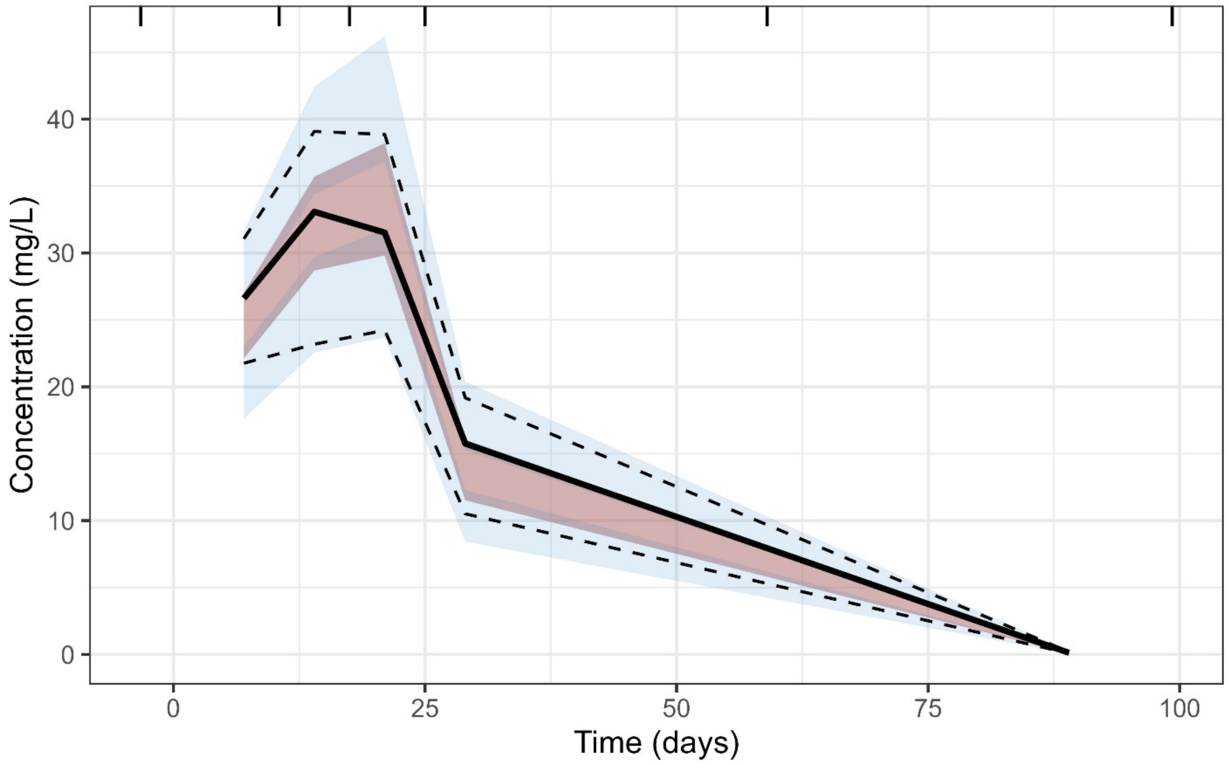

(C) Skin parasite score

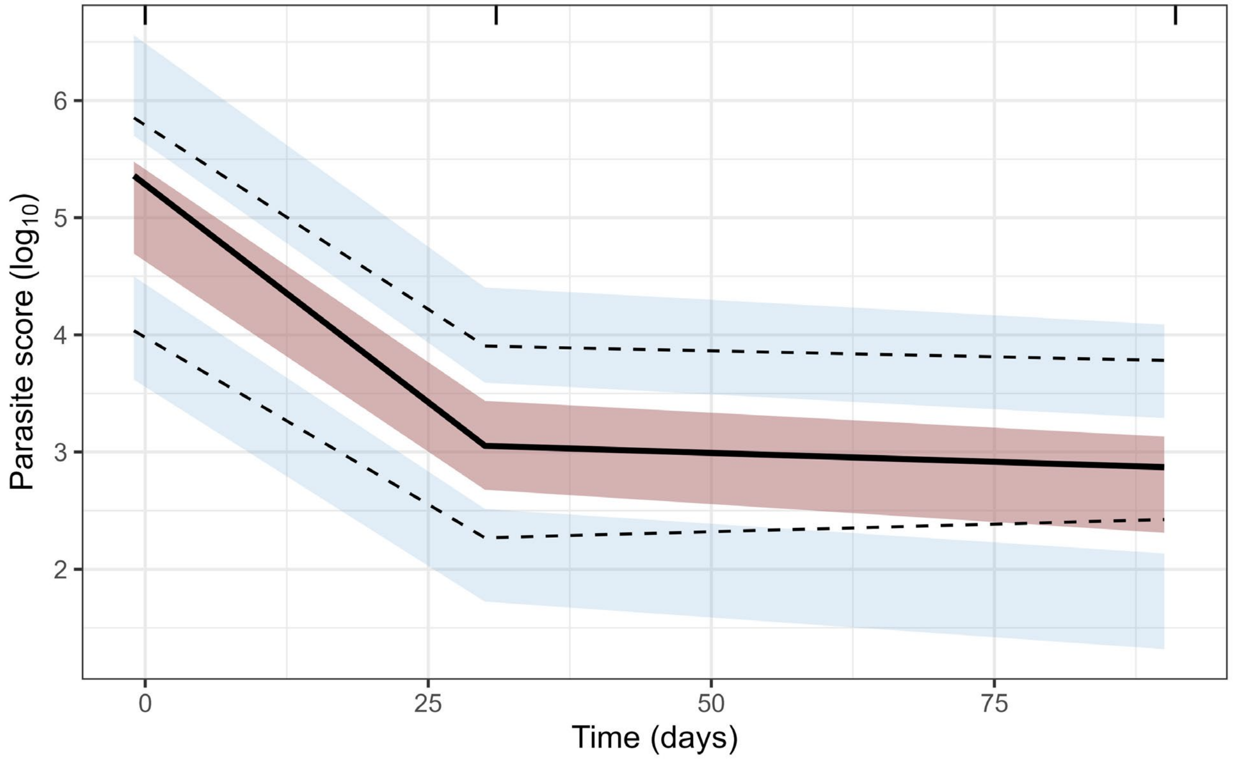

References

1. Verma, S. *et al.* Quantification of parasite load in clinical samples of leishmaniasis patients: IL-10 level correlates with parasite load in visceral leishmaniasis. *PLoS One* **5**, e10107 (2010).

2. Hossain, F. *et al.* Real-time PCR in detection and quantitation of Leishmania donovani for the diagnosis of Visceral Leishmaniasis patients and the monitoring of their response to treatment. *PLoS One* **12**, e0185606 (2017).

3. Chu, W.-Y. *et al.* Disease-Specific Differences in Pharmacokinetics of Paromomycin and Miltefosine Between Post-Kala-Azar Dermal Leishmaniasis and Visceral Leishmaniasis Patients in Eastern Africa. *J. Infect. Dis.* **00**, (2024).
